# Seroprevalence of SARS-CoV-2, Symptom Profiles and Sero-Neutralization in a Suburban Area, France

**DOI:** 10.3390/v13061076

**Published:** 2021-06-04

**Authors:** Anne Gégout Petit, Hélène Jeulin, Karine Legrand, Nicolas Jay, Agathe Bochnakian, Pierre Vallois, Evelyne Schvoerer, Francis Guillemin

**Affiliations:** 1 IECL, Université de Lorraine, CNRS, Inria, F-54000 Nancy, France; anne.gegout-petit@univ-lorraine.fr (A.G.P.); pierre.vallois@univ-lorraine.fr (P.V.); 2 LCPME, Université de Lorraine, CNRS, F-54000 Nancy, France; e.schvoerer@chru-nancy.fr; 3 Laboratoire de Virologie, CHRU de Nancy Brabois, F-54500 Vandoeuvre-lès-Nancy, France; 4 CIC Epidémiologie Clinique, CHRU-Nancy, Inserm, Université de Lorraine, F-54000 Nancy, France; k.legrand@chru-nancy.fr (K.L.); a.bochnakian@chru-nancy.fr (A.B.); francis.guillemin@chru-nancy.fr (F.G.); 5 LORIA, CHRU-Nancy, Université de Lorraine, CNRS, Inria, LORIA, F-54000 Nancy, France; nicolas.jay@univ-lorraine.fr

**Keywords:** COVID-19, seroprevalence, symptoms profile, precariousness

## Abstract

The World Health Organisation recommends monitoring the circulation of severe acute respiratory syndrome coronavirus 2 (SARS-CoV-2). We investigated anti–SARS-CoV-2 total immunoglobulin (IgT) antibody seroprevalence and in vitro sero-neutralization in Nancy, France, in spring 2020. Individuals were randomly sampled from electoral lists and invited with household members over 5 years old to be tested for anti–SARS-CoV-2 (IgT, i.e., IgA/IgG/IgM) antibodies by ELISA (Bio-rad); the sero-neutralization activity was evaluated on Vero CCL-81 cells. Among 2006 individuals, the raw seroprevalence was 2.1% (95% confidence interval 1.5 to 2.9), was highest for 20- to 34-year-old participants (4.7% (2.3 to 8.4)), within than out of socially deprived area (2.5% vs. 1%, *p* = 0.02) and with than without intra-family infection (*p* < 10^−6^). Moreover, 25% of participants presented at least one COVID-19 symptom associated with SARS-CoV-2 positivity (*p* < 10^−13^), with highly discriminant anosmia or ageusia (odds ratio 27.8 [13.9 to 54.5]); 16.3% (6.8 to 30.7) of seropositive individuals were asymptomatic. Positive sero-neutralization was demonstrated in vitro for 31/43 seropositive subjects. Regarding the very low seroprevalence, a preventive effect of the lockdown in March 2020 can be assumed for the summer, but a second COVID-19 wave, as expected, could be subsequently observed in this poorly immunized population.

## 1. Background

The World Health Organisation (WHO) [1] recommends a close observation of the circulation of the severe acute respiratory syndrome coronavirus 2 (SARS-CoV-2), including local seroprevalence surveys, to adapt the public health response to COVID-19 [2]. Indeed, population containment, sanitary procedures and planning must be defined in terms of a quantified health concern. To estimate the proportion of individuals who were or are infected by the virus, serology assays for detecting anti-SARS-CoV-2 antibodies are useful in all individuals with mild (or no) clinical signs, with or without a RT-PCR test.

Between January and July 2020, 13 general-population serology surveys of SARS-CoV-2 were reported in Europe, 10 in the United States, four in Brazil, one in Pakistan and one in Japan (personal communication). Most (*n* = 20) estimated the seroprevalence between 0–5%; half under 2.5%. Six studies conducted in regions highly affected by the epidemic estimated the anti–SARS-CoV-2 antibody seroprevalence at more than 15% [2,3,4,5,6,7]. Few studies investigated the relation between seroprevalence and social precariousness, despite some evidence that health inequalities are reflected in the pandemic [8,9].

Serology assays usually detect antibodies against the viral spike “S” and nucleocapsid “N” protein, both being highly antigenic and widely expressed during SARS-CoV-2 infection [10]. After primary infection, immunoglobulin G (IgG) levels increase continuously, peaking at about 6 weeks after infection and often remaining high for 6 months. The neutralizing activity usually peaks after 4 weeks and then can slowly decrease [11]. At 4 months after a first positive anti–SARS-CoV-2 antibody result, 41% of infected patients become negative for anti-N antibodies, but most are still positive for anti-S antibodies [12]. Viral infection requires the receptor-binding domain (RBD) of the S protein, which is the molecular determinant of viral attachment to the host cell receptor angiotensin-converting enzyme 2 (ACE-2) [13]. Antibodies targeting the S protein neutralize the virus entering into the cell, and the IgM, IgA and IgG antibodies directed at the RBD of the S protein are highly neutralizing [14,15,16]. Thus, well-standardized, reproducible antibody assays are crucial to establish correlates of risk and protection so that SARS-CoV-2 neutralization assays can be used for antibody monitoring in natural infection and vaccine trials [17].

The first COVID-19 cases were reported in France in January 2020 [18] and a strong SARS-CoV-2 emergence was observed in northeast France in March and April 2020, with numerous patients presenting at Nancy University hospital, France [19]. To document the strength of SARS-CoV-2 circulation, biological samples from a random sample of the population were needed for serological testing [1]. Our primary objective was to estimate the anti–SARS-CoV-2 total Ig (IgT) antibody seroprevalence in a random sample of the population of the Grand Nancy Metropolitan area. The secondary objectives were to estimate (1) the proportion of asymptomatic cases or symptom profiles, (2) the proportion of seropositive people according to level of social precariousness, and (3) the in vitro neutralization capacity of viral infectivity for the detected anti–SARS-CoV-2 antibodies.

## 2. Materials and Methods

The COVAL Nancy cross-sectional study was conducted between 26 June and 24 July 2020.

### 2.1. Sampling

The target population consisted of all inhabitants of the Grand Nancy metropolitan area who were ≥5 years old on 1 June 2020. Adults randomly sampled from the electoral lists were invited to participate with all household members. To ensure representativeness, sampling was carried out by strata of homogeneous housing areas according to socio-economic criteria (IRIS habitat; INSEE Source(s): INSEE, Géographie à l’infra-communale (Official Geographic Code)), with each homogeneous housing area (IRIS) associated with the European deprivation index (EDI) [20]. This continuous index consists of ecological variables best identified to reflect the individual experience of deprivation and is grouped by INSEE into classes by quintiles; 5 is the most deprived class.

From preliminary regional estimates with strong county disparities [21] and given serologic test sensitivity (100%) and specificity (99.5%), with 1% target precision and 95% confidence interval, we needed 1987 individuals to detect a 5% seroprevalence. Accordingly, the survey logistics were organized to account for estimated individual response rate, household members’ participation, appointment attendance and agreeing to blood sampling; we aimed to include 2000 individuals.

All invited individuals were informed of the objectives and the workflow of the study by using comprehensive messaging adapted to age. All individuals gave their signed consent.

Ethical approval was obtained (Comité de Protection des Personnes EST III, NANCY, France: ID RCB 2020-A01593-36) on 16 June 2020 and from the French Commission for Individual Data Protection and Public Liberties (CNIL) on 19 June 2020.

During the inclusion visit to the Nancy University Hospital, each participant completed a self-reporting questionnaire adapted to age (adult, adolescent; child questionnaire completed by parents). The following data were collected:

– socio-demographic characteristics: age, sex, socio-professional category, education level;
– Evaluation of Deprivation and Inequalities in Health Examination Centres (EPICES) questionnaire (for adults) [22], a composite index commonly used to measure individual deprivation. A score is calculated on the basis of 11 weighted questions related to material and social deprivation, ranging from 0 to 100 (>30 associated with social deprivation).
– health characteristics: body mass index, smoking status, influenza vaccination, health problems, pregnancy;
– potential contacts with a person with COVID-19: perception of infection with the virus, relatives infected;
– symptoms experienced since mid-February: fever, cough, runny nose, chest pain, anosmia or ageusia, sore throat, muscle pain, aches, fatigue, headaches, skin rashes, appetite loss, shortness of breath, diarrhea, loss of balance, abdominal pain, nausea, and irritated eyes. According to the European Center for Disease Prevention and Control [23], at least one symptom among fever, cough, anosmia or ageusia, and shortness of breath indicates COVID-19.

### 2.2. Serology

Blood samples were centrifuged to collect serum, which was stored at +4 °C and then −20 °C. Total anti-SARS-CoV-2 antibodies (IgA/IgG/IgM) were detected by using ELISA (Platelia SARS-CoV-2 Total Ab Assay ref 72710 Bio-rad, Marnes-la-Coquette, France) on an Evolis Premium device (Bio-rad, Marnes-la-Coquette, France); LOD = 0.320; LOS = 0.533. IgM and IgG antibodies were detected by using an immuno-chromatographic test (BIOSYNEX COVID-19 BSS, Ref. SW40005, Biosynex, Illkirch-Graffenstaden, France), with IgA by ELISA (Anti-SARS-CoV-2 Assay, Euroimmun, Bussy Saint Martin, France) on SARS-CoV-2–seropositive samples and on samples from SARS-CoV-2–seronegative individuals living with SARS-CoV-2–positive individuals.

**Figure 1:**
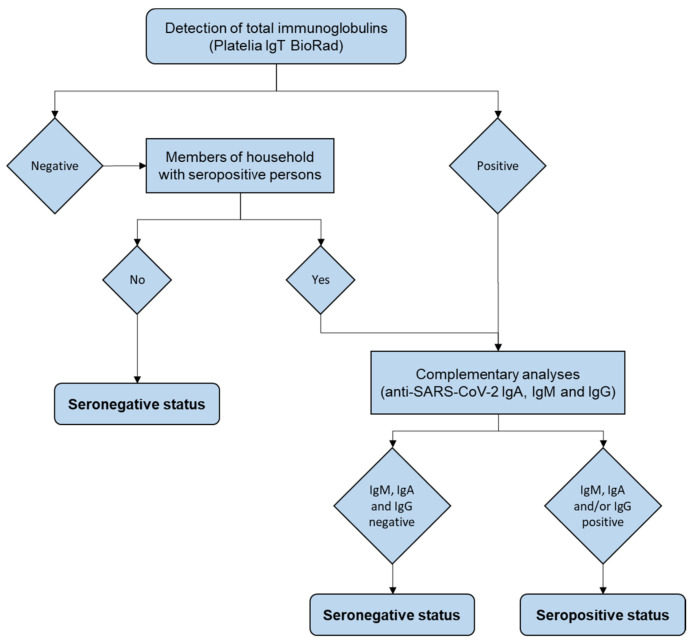
Flow of serology testing in the study.

A person was classified as SARS-CoV-2–seropositive if at least two serology tests were positive (Figure 1).

### 2.3. Microneutralization Assay

Microneutralization assay was realized with a reference virus (D614G strain), in BSL3. The SARS-CoV-2 strain from a positive respiratory sample (Covi-Lor collection, Nancy University hospital, Nancy, France) was cultured on Vero CCL-81 cells (provided by L2CM Laboratory, Vandoeuvre les Nancy, France). Sera positive for anti-SARS-Cov-2 antibodies were diluted from 1/10 to 1/640 and incubated with virus suspension for 2 h. Cells were inoculated with the final suspension. Each dilution was tested five times in the same experiment and each sample in two independent experiments. The cytopathic effect was read on day +6.

Negative controls were uninfected cells; positive controls were the virus incubated without sera and the virus incubated with SARS-CoV-2–negative sera at a 1/10 ratio.

The samples were classified according to neutralization activity at 1:40 dilution: neutralization > 50% (NT50).

### 2.4. Statistical Analysis

All statistical analyses involved using R Core Team (2020). (R: A language and environment for statistical computing. R Foundation for Statistical Computing, Vienna, Austria. 3.6.0). To calculate the 95% confidence interval for fractions, we used the normal approximation interval except for the Clopper Pearson exact method based on binomial distribution for the SARS-CoV-2–positive sample (too small in size). The raw seroprevalence estimate was adjusted for age, sex, and EDI quintile, then standardized to the metropolitan and national population [24]. For comparing seroprevalence or characteristics between groups, we used chi-square or Fisher exact test and logistic regression, estimating odds ratios (ORs) and 95% confidence intervals (CIs). We used the R package ClustOfVar, Université de Bordeaux, Bordeaux, 1.1 [25] to study the clustering of symptom variables and to draw dendograms. Intra-household infection spread was tested by a permutation test [26]. The principle was to generate, by simulation, the empirical distribution of the number of infected households under the null hypothesis, respecting the number of individual cases and the structure of the households observed in the sample. We used simulation to calculate the relative risk (and 95% CI) of being SARS-CoV-2–positive in a household with a SARS-CoV-2–positive member.

## 3. Results

### 3.1. Sample Description 

**Figure 2:**
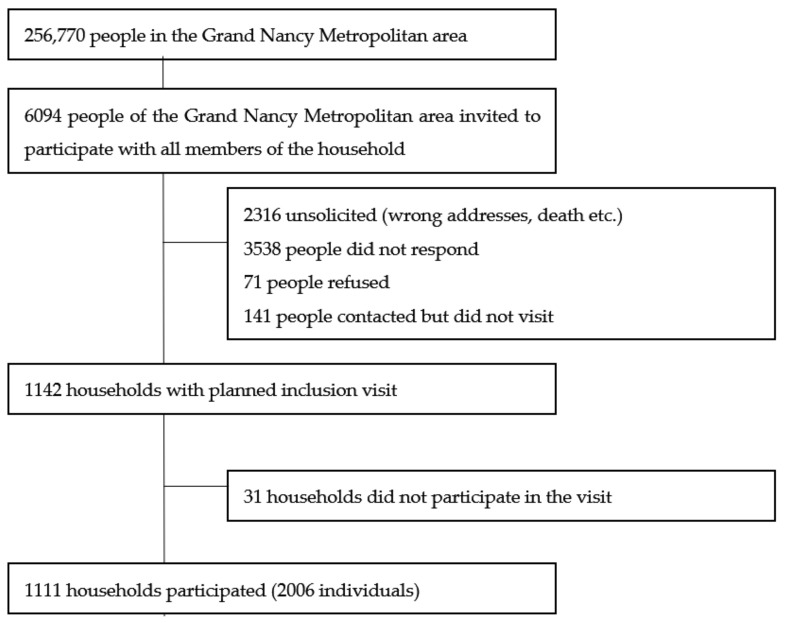
Flow of participants in the study.

We invited 6094 people to participate in order to enable the inclusion of 2006 participants, aged 5 to 95 years old from 1111 households (Figure 2): 55% were women and 148 under 18-year-old; 469 people came to the visit alone, 938 came as a couple and the others (599) came as a family of three to six people.

The Grand Nancy Metropolitan area comprises 110 IRIS zones; 108 were represented. People in neighborhoods with a high socio-economic level (measured by the EDI) and high socio-professional category responded better than others. According to the EPICES score, 388 of the 1816 (21%) participants with this score were considered to live in socially precarious situations.

Social precariousness was also linked to the IRIS EDI quintile: less than 16% in the first three quintiles, up to 23% in the fourth quintile and 40% in the last quintile (*p* < 10^−5^); it also increased with age: 16% in the 5–44 age group, 18% in the 45–64 age group and 28% in those over 65 (*p* < 10^−6^).

Among the 2006 participants, 16% were smokers, 2% used nicotine substitutes, and 29% were former smokers. Moreover, 294 (14.6%) reported at least one comorbidity (among: hypertension, cancer, diabetes, kidney failure, liver problems, immune deficiency, immunosuppressive therapy, severe obesity). The presence of a comorbidity was not related to EDI score but was strongly related to social precariousness: 26% of those in precarious situations had at least one comorbidity as compared with 13% of others (*p* < 10^−9^).

In total, 252 (12.6%) participants thought they were infected with COVID-19 because they experienced symptoms (86%) and/or had been in contact with a sick person (44%). Among contacts with COVID-19, 42% were from work, 28% were family and 22% were friends.

### 3.2. General Seroprevalence

According to the results of anti-SARS-CoV-2 IgT detection and complementary analyses performed as described in Figure 1, 43 of the 2006 participants were found to be seropositive. Thus, seroprevalence was 2.1% (95% CI 1.5 to 2.8). On adjustment for age, sex and EDI quintile, seroprevalence was 2.5 (1.8 to 3.3) standardized for the Grand Nancy Metropolitan area and 2.3 (1.7 to 3.1) standardized for France.

Among the 43 SARS-CoV-2–positive samples, none was positive for anti–SARS-CoV-2 IgM antibody only, 17 (39.5%) were positive for anti–SARS-CoV-2 IgM and IgG antibodies and 26 (60.5%) were positive for anti–SARS-CoV-2 IgG antibody only (Table 1).

### 3.3. Seroprevalence by Age and Socioeconomic Status 

Seroprevalence was highest in the 20–34 age group (4.7% (95% CI 2.3 to 8.4)) and in people from areas of lower socioeconomic level (2.7% vs. 1% for EDI quintiles 3, 4 and 5 vs. 1 and 2, *p* = 0.02). We observed little difference in prevalence among people without and with a baccalaureate diploma (1.4% vs. 2.6%, *p* = 0.10) and social precariousness (1.0% vs. 2.5% for EPICES scores > 30 vs. ≤ 30, *p* = 0.09). Social precariousness had no protective effect as seen by the probability of precariousness estimated with adjusted or random-effects models (*p* = 0.07 to 0.11) (see Table 2 for one of the models).

### 3.4. Seroprevalence with Other Factors

Seroprevalence did not differ between smokers and non-smokers (1.2% vs. 2.4%) (*p* = 0.18) or by sex, household size, weight status or presence of risk factors (Table 2). Of the 252 participants who thought they had COVID-19, 31 (12%) were SARS-CoV-2–positive and 6 (2%) were negative but lived in a household with a SARS-CoV-2–positive person. Furthermore, 72% of SARS-CoV-2–positive individuals thought they had been infected. Among those who did not think they had COVID-19, 12 (0.7%) were SARS-CoV-2–positive and 20 (1.1%) were negative but lived in a household with a SARS-CoV-2–positive person.

For households, 34 (3.1% (95% CI 2.1 to 4.3)) had at least one SARS-CoV-2–positive individual. Household seroprevalence was slightly higher if the household size was ≥3 (4.1% vs. 2.7%, *p* = 0.26). We found intra-household spread (permutation test, *p* < 10^−6^), and probability of infection was multiplied by 30 (95% CI 11 to 78) with a SARS-CoV-2–positive household member.

### 3.5. Symptoms

In the overall sample, 25% (95% CI 23 to 27) showed symptoms that would indicate they had COVID-19. This criterion was related to seroprevalence (6.5% vs. 0.7% with and without COVID-19 symptoms, *p* < 10^−13^). Nearly half of the individuals (47%) reported experiencing at least one of the 18 collected symptoms (14% one “intense” symptom). Seroprevalence was higher with than without at least one symptom (3.8% vs. 0.7%, *p* < 10^−5^) and when at least one of the symptoms was qualified as “intense” (9.4% vs. 0.7%, *p* < 10^−17^). For each of the identified symptoms (except irritated eyes and rash), seroprevalence was higher when the symptom was expressed (see Table 3), with anosmia or ageusia the most discriminating symptom (OR 27.8 [95% CI 13.9 to 54.5]).

Focusing on the 43 SARS-CoV-2–positive individuals, 7 (16.3% (95% CI 6.8 to 30.7)) experienced no symptoms. The asymptomatic form did not depend on age (*p* = 0.4) or sex (*p* = 0.9).

**Figure 3:**
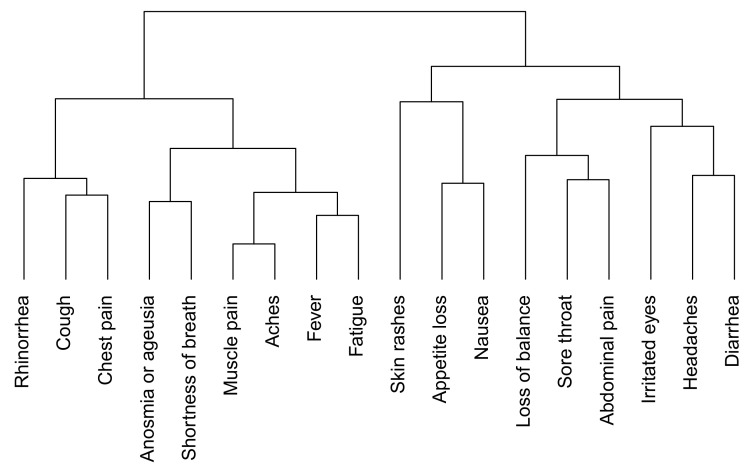
Dendogram of symptoms in seropositive individuals (*n* = 43).

Most symptomatic people experienced symptoms in March (Figure 3), which shows a clear effect of confinement on slowing/stopping the spread of the disease.

**Figure 4:**
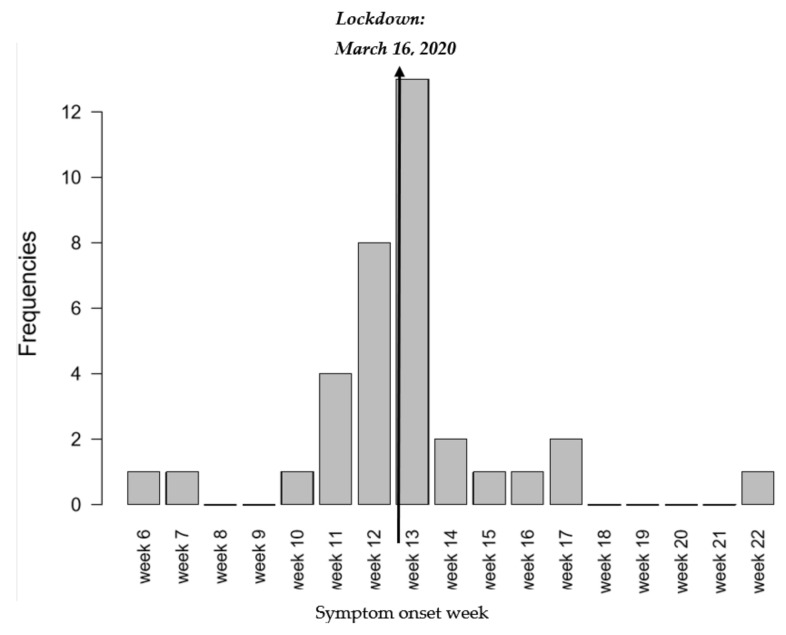
Date of onset of symptoms in seropositive symptomatic individuals (*n* = 36).

Among those with at least one symptom, many (72%) reported this symptom as “intense”. A cluster analysis of symptoms in SARS-CoV-2–positive individuals grouped anosmia or ageusia with influenza-like illness (Figure 4). Anosmia or ageusia was strongly associated with shortness of breath (*p* = 0.0002) and fever (*p* = 0.0008) but almost never occurred without fever (1/17).

### 3.6. Seroneutralization Assay

For 31/43 (72% [95% CI 56 to 85]) SARS-CoV-2–positive individuals, antibody detection was associated with neutralization activity (NT50 ≥ 40) (Table 1). Twelve seropositive samples presented no or weak neutralization capacity. Among 17 serum samples with recent seroconversion (IgM+IgG positive), 12 (71%) presented sero-neutralization activity, as did 19/26 (73%) with older seroconversion (IgG only) (NT50 ≥ 40). Seroconversion did not depend on age (*p* = 0.8), sex (*p* = 0.6), or time between symptoms and data collection. Sero-neutralization did not depend on the presence of symptoms (*p* = 0.9). The NT50 was increased with strong symptoms (*p* = 0.24).

### 3.7. Consequences of Low Anti-SARS-CoV-2 Prevalence on the Epidemic Evolution

At the end of the first epidemic peak, the seroprevalence of SARS-CoV-2 (i.e., 2.1%) was very low, associated with a low prevalence of sero-neutralization, highlighting a poorly immunized population.

**Figure 5:**
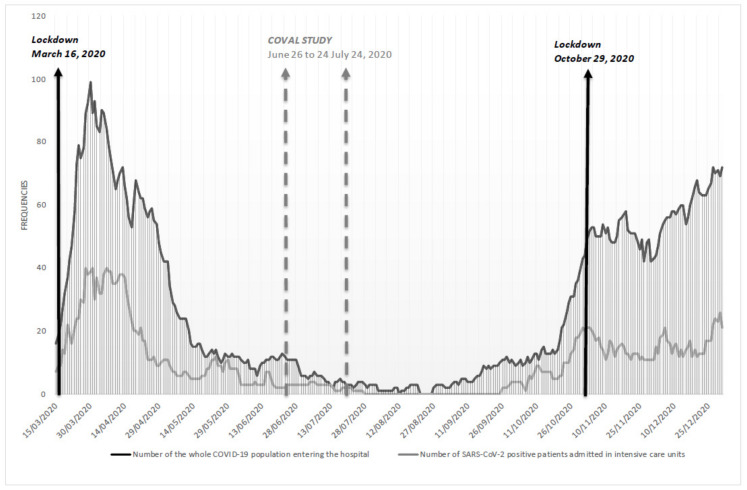
Number of SARS-CoV-2 positive patients from the Grand Nancy metropolitan at the Nancy University Hospital between 15 March and 31 December 2020 (black: all, grey: intensive care units).

As expected, the metropolitan population was highly susceptible to the second epidemic wave. Data from the Regional University Hospital displayed an increase in the number of the whole COVID-19 population entering the hospital and also an increase in the number of SARS-CoV-2-positive patients admitted in intensive care units. (Figure 5).

## 4. Discussion

The anti-SARS-CoV-2 seropositivity of the population from the Grand Nancy Metropolitan area was evaluated by using the detection of specific antiviral IgT.

Within the Coronaviridae family, SARS-CoV-2 is phylogenetically close to SARS-CoV and to a lesser extent MERS-CoV [27]. Cross-reactivity with seasonal coronaviruses has to be considered. Even if recently, pre-existing HCoV-NL63 antibody response cross-reacting with some SARS-CoV-2 antigens was detected in both pre- and mid-pandemic infected individuals [28], though no cross-reactivity of serology methods with the seasonal coronavirus was reported [29] and no neutralizing activity of SARS-CoV-2 in pre-pandemic sera was observed [30]. All IgT-positive serum samples were positive for antibodies against the RBD of S protein (Biosynex COVID19 BSS). IgM and IgG antibodies directed against the RBD are assumed to decrease in titers during the 6 months post-infection. Since the study took place less than 6 months after the first epidemic peak, the seroprevalence is representative of the exposure of the population to SARS-CoV-2 during the first epidemic peak in the North-East of France. The question of the remaining humoral immunity is currently unsolved, but even though the IgG titers and neutralizing activity decreases, the number of RBD-specific memory B cells was unmodified at 6 months, which can contribute to the immune response to a secondary infection [31]. Moreover, the presence of high IgG and IgM antibodies to the spike S1 C-terminal domain in recovered patients might be associated with efficient immune protection in COVID-19 patients [32]. Therefore, we can assume that IgT-positive individuals in the study may be protected against COVID-19 if exposed during the second epidemic peak.

A recent work reported that standard commercially available SARS-CoV-2 IgG results could be a useful surrogate for neutralizing antibody testing [32]. However, in the present study, antibody neutralizing titers were determined in vitro by using a native SARS-CoV-2 strain in order to be closer to the physiological infection of cells by SARS-CoV-2 in northeast France [33]. The total of 12/43 SARS-CoV-2–positive individuals presenting no or weak neutralizing titers agrees with a study of COVID-19 recovered patients [33].

From an epidemiological point of view, seropositivity was associated with the ecological marker of social deprivation (EDI) but not when assessed with social precariousness at the individual level (EPICES), which may indicate some protective effect. Such discrepancy might be related to the social isolation of deprived individuals during the lockdown, independent in terms of residence, potentially resulting in less exposure for many reasons (unemployment, fear of meeting people).

In July 2020, the low seroprevalence of SARS-CoV-2 (i.e., 2.1% SARS-CoV-2–positive cases among the 2006 included individuals, with one in six SARS-CoV-2–positive individuals who remained asymptomatic) underlined how the Grand Nancy Metropolitan area population remained immuno-naïve and susceptible to the second epidemic wave that occurred during autumn/winter 2020/2021 in France. Moreover, all IgT-positive individuals did not show a sufficient in vitro neutralization titer. Consequently, and as expected, by the end of 2020, both the number of SARS-CoV-2 positive patients admitted in intensive care units and the number of the whole COVID-19 population entering the University hospital of Nancy rose quickly from the beginning of October up to December 2020 (Figure 5), confirming the urgent need for collective immunity thanks to vaccination. Vaccination has been applied from January 2020 in France and almost a third of the population of the area has been vaccinated in May. (fr.Statista.com). Thus, such an identical study is no longer feasible, because distinction of post-infectious and post-vaccination immunity would be needed with at least two different serological methods. In our results, the number of seropositive individuals is actually low but we collected information on the viral circulation and on risk factors of infection allowing us to understand the progression of the first epidemic wave.

To put our results into a pandemic historical perspective, updating our literature survey until end of March 2021 identified another 35 general population serology surveys, including 11 in Europe, 10 in Asia and nine in the USA, with heterogenous estimates over time and space (personal communication). Most of the seroprevalence estimates were below 10%, but increased to over 20% in Sudan (22.3%) [34] and South Africa (23.7% to 63%) [35,36].

This study has strengths. First, in this relatively small geographical area, we were able to stratify sampling based on homogenous population zones (IRIS zones) and tag the level of social deprivation by using the corresponding ecological EDI index, to better represent the target population in terms of this variable that has been considered an important risk factor for COVID-19 (8). Second, sero-prevalent cases were carefully identified by using several methods for confirming seropositivity (Figure 1). Third, sero-neutralization capacity was investigated in duplicate, in line with recommended standard practices, instead of derived from IgG results [33,37]. Microneutralization was realized by using the reference-virus, D614G strain, in BSLN3. At that time, this was the main SARS-CoV-2 strain circulating in France. Nowadays, many variants of concern have emerged and spread. The evaluation of sero-neutralization capacities is more complex since some strains (i.e., the 20H/501Y.V2 “South African” variant, lineage B1.351) partially escape to humoral immunity [38].

The study has some limitations. First, the sampling electoral database, chosen for its immediate availability in such a small and delimited area, did not completely cover the adult population because some people left the area (among the 6094 people invited, 38% were not properly solicited (wrong addresses, death etc.)), newcomers to the area had not yet registered (not mandatory), and non-European citizens were not eligible, which creates some representativeness bias [39]. Second, the estimated response rate was relatively low in a period immediately following the lockdown, with many people already gone away for July summer holidays. Third, all data were self-reported, which may lead to some measurement (declaration) bias. Moreover, even if the number of individuals was sufficient to satisfy the main objective, the study lacked statistical power for risk factors.

Finally, with novel SARS-CoV-2 variants emerging all over the world [40], the neutralizing activity of positive sera against novel SARS-CoV-2 variants should be evaluated. As the virus has shown the ability to acquire mutations, changing its susceptibility to antibodies and immunity, either after natural infections or vaccination, the seric neutralizing ability has to be regularly reevaluated in various human population typologies and against successive SARS-CoV-2 variants of concern or interest [41].

In conclusion, IgT assays are key tools to monitor the circulation of SARS-CoV-2 and the impact of public health guidelines [42]. In this population of low anti-SARS-CoV-2 seroprevalence, a beneficial effect of the lockdown on virus circulation can be assumed. IgT seroprevalence was higher for young adults and was associated with intra-family SARS-CoV-2 transmission. Of course, the spread of the pandemic is complex, its dynamics, measured by its R0, are constantly modified by the increase in the number of people who have contracted the disease, by vaccination, sanitary restrictions, but also the emergence of more or less transmissible variants. Such a study provides historical data on epidemics to prepare for future pandemics.

## Figures and Tables

**Table 1:** Detection of anti-SARS-CoV-2 total immunoglobulin (IgT) antibodies, determination of Ig isotypes and sero-neutralization of seropositive sera.

*n*	IgT	IgM	IgA	IgG	Serological Status	Seroneutralization Capacity (*n*)
1923	Negative	NR	NR	NR	Seronegative	NE
32	Negative	Negative	Negative	Negative	Seronegative *	NE
8	Positive	Negative	Negative	Negative	Seronegative	NE
2	Negative	Negative	Positive	Positive	Seropositive *	0
14	Positive	Positive	Positive	Positive	Seropositive	11
0	Positive	Positive	Positive	Negative	Seropositive	NE
3	Positive	Positive	Negative	Positive	Seropositive	1
17	Positive	Negative	Positive	Positive	Seropositive	15
0	Positive	Positive	Negative	Negative	Seropositive	NE
0	Positive	Negative	Positive	Negative	Seropositive	NE
7	Positive	Negative	Negative	Positive	Seropositive	4
2006	Total					

* Living with seropositive people; NE: not evaluated.

**Table 2:** Number of cases, seroprevalence for each risk factor modality. Data are 95% confidence intervals (CIs), odds ratios (ORs) and *p* values. (Bold indicate significance at 0.05 level).

Modalities	Positive/Total	%	% CI	OR	OR CI	*p*
Age						
05–19	2/203	1.0	0.1–3.5	0.7	0.1–3.2	0.65
20–34	10/215	4.7	2.3–8.4	3.4	1.2–10.9	**0.03**
35–49	5/350	1.4	0.5–3.3	ref		
50–64	16/553	2.9	1.7–4.7	2.1	0.8–6.3	0.16
65–79	9/573	1.6	0.7–3.0	1.1	0.4–3.6	0.86
80+	1/112	0.9	0.0–4.9	0.7	0.0–3.9	0.67
Gender						
Female	24/1104	2.2	1.4–3.2	ref		
Male	19/902	2.1	1.3–3.3	1.0	0.5–1.8	0.92
Quintile EDI						
1–2	6/615	1.0	0.4–0.2	ref		
3–4-5	37/1391	2.7	1.9–3.7	2.8	1.3–7.3	**0.02**
Household Size						
1	9/364	2.5	1.1–4.6	ref		
2	19/882	2.2	1.3–3.3	0.9 *	0.4–2.0	0.74
>=3	15/760	2.0	1.1–3.2	0.6	0.2–1.5	0.24
Educational Level: Baccalaureate						
Yes	33/1266	2.6	1.8–3.6	ref		
No	8/586	1.4	0.6–2.7	0.5	0.2–1.1	0.1
missing	154					
Smoking status						
Non-Smoker	38/1583	2.4	0.2–3.3	ref	ref	
Smoker	4/338	1.2	0.3–3.0	0.5	0.1–1.2	0.17
Missing	85					
Body Mass Index						
<25	22/1162	1.9	1.2–2.9	ref		
25–30	15/551	2.7	1.5–4.5	1.5	0.7–2.8	0.27
>=30	6/284	2.1	0.8–4.5	1.1	0.4–2.6	0.81
missing	9					
Comorbidity						
No	38/1726	2.20	1.6–3.0	ref		
Yes	5/280	1.78	0.6–4.1	0.8	0.3–1.9	0.65
Precariousness (Adjusted on Age, Sex and EDI)						
EPICES <= 30	35/1428	2.5	1.7–3.4	ref		
EPICES > 30	4/388	1.0	0.3–2.6	0.4	0.1–1.0	0.1

**Table 3:** Frequency of symptoms by serology status.

Clinical Criterion	Seropositive	Seronegative	*p*-Value
*n*	43	2006	
At least one symptom	% 83.7	% 47.6	3 × 10^−6^
At least one intense symptom	60.5	13.1	1 × 10^−18^
Clinical criteria poss Covid-19 *	74.4	23.8	3 × 10^−14^
Fever	62.8	14.7	1 × 10^−17^
Cough	53.5	12.1	1 × 10^−15^
Fatigue	48.8	10.9	6 × 10^−11^
Shortness of breath	46.5	6.6	6 × 10^−13^
Aches	41.9	8.2	2 × 10^−14^
Anosmia/ageusia	39.5	2.3	5 × 10^−44^
Muscle pain	37.2	10.4	3 × 10^−8^
Sore throat	34.9	14.7	3 × 10^−4^
Headaches	32.6	10.1	2 × 10^−6^
Rhinorrhea	30.2	16.6	0.02
Chest pain	25.6	6.3	6 × 10^−17^
Diarrhea	23.30	8.4	0.0006
Abdominal pain	20.9	6.8	0.0004
Loss of balance	14.0	4.0	0.001
Nausea	14.0	3.8	0.0009
Appetite loss	11.6	1.1	2 × 10^−9^
Skin rashes	7.0	4.9	0.52
Irritated eyes	4.7	6.0	0.70

* Definition of ECDC.

## Data Availability

The data for this article will be shared on reasonable request to the corresponding author.
